# The *Arabidopsis thaliana* N‐recognin E3 ligase PROTEOLYSIS1 influences the immune response

**DOI:** 10.1002/pld3.194

**Published:** 2019-12-26

**Authors:** Christopher J. Till, Jorge Vicente, Hongtao Zhang, Maria Oszvald, Michael J. Deery, Victoria Pastor, Kathryn S. Lilley, Rumiana V. Ray, Frederica L. Theodoulou, Michael J. Holdsworth

**Affiliations:** ^1^ School of Biosciences University of Nottingham Loughborough UK; ^2^ Plant Sciences Department Rothamsted Research Harpenden UK; ^3^ Cambridge Centre for Proteomics Department of Biochemistry University of Cambridge Cambridge UK; ^4^ Área de Fisiología Vegetal Departamento de Ciencias Agrarias y del Medio Natural Universitat Jaume I Castellón Spain

**Keywords:** N‐degron pathway, pipecolic acid, plant immunity, priming, proteostasis, PRT1

## Abstract

N‐degron pathways of ubiquitin‐mediated proteolysis (formerly known as the N‐end rule pathway) control the stability of substrate proteins dependent on the amino‐terminal (Nt) residue. Unlike yeast or mammalian N‐recognin E3 ligases, which each recognize several different classes of Nt residues, in *Arabidopsis thaliana*, N‐recognin functions of different N‐degron pathways are carried out independently by PROTEOLYSIS (PRT)1, PRT6, and other unknown proteins. PRT1 recognizes type 2 aromatic Nt‐destabilizing residues and PRT6 recognizes type 1 basic residues. These two N‐recognin functions diverged as separate proteins early in the evolution of plants, before the conquest of the land. We demonstrate that loss of PRT1 function promotes the plant immune system, as mutant *prt1‐1* plants showed greater apoplastic resistance than WT to infection by the bacterial hemi‐biotroph *Pseudomonas syringae* pv *tomato* (*Pst*) DC3000*.* Quantitative proteomics revealed increased accumulation of proteins associated with specific components of plant defense in the *prt1‐1* mutant, concomitant with increased accumulation of salicylic acid. The effects of the *prt1* mutation were additional to known effects of *prt6* in influencing the immune system, in particular, an observed over‐accumulation of pipecolic acid (Pip) in the double‐mutant *prt1‐1 prt6‐1*. These results demonstrate a potential role for PRT1 in controlling aspects of the plant immune system and suggest that PRT1 limits the onset of the defense response via degradation of substrates with type 2 Nt‐destabilizing residues*.*

## 1 INTRODUCTION

**Figure 1:**
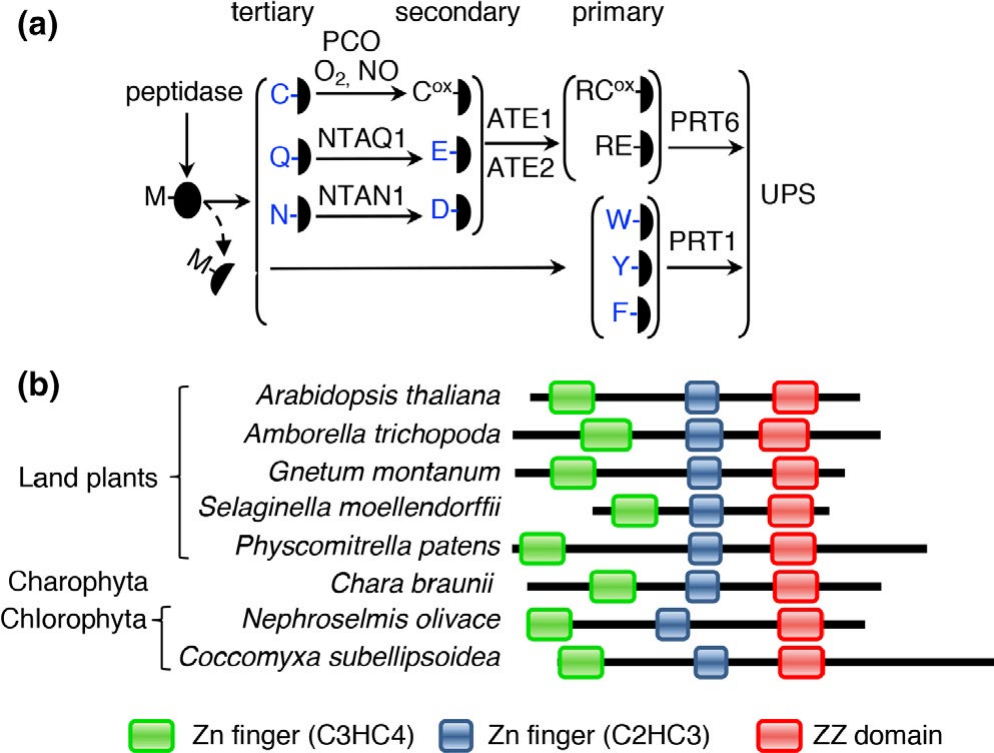
Structures of PRT1‐like proteins in plants. (a) Schematic of the arabidopsis PRT6‐ and PRT1 N‐degron pathways. Single letter codes for residues are shown. PRT1, PROTEOLYSIS1; PRT6, PROTEOLYSIS6; ATE, arginyl‐tRNA protein transferase; NTAN, Nt‐Asn amidase; NTAQ, Nt‐Gln amidase; PCO, PLANT CYSTEINE OXIDASE; UPS, Ubiquitin Proteasome System. Black ovals represent protein substrates. Experimentally proven destabilizing residues in plants are shown in blue (Millar et al., 2019). (b) Schematic alignment of PRT1‐like sequences in plant taxa. PRT1‐like proteins were defined as containing two Zn‐RINGs and a ZZ domain. Green boxes, Zn finger (C3HC4); blue boxes, Zn finger (C2HC3); red boxes, ZZ domain

The N‐degron pathways (previously called the “N‐end rule pathway”) regulate the half‐life of diverse cellular proteins. This is achieved by recognition of amino‐terminal (Nt‐) residues of proteins as part of N‐degrons, where one or more lysine residues are available for ubiquitylation and the N terminus is accessible (Gibbs, Bacardit, Bachmair, & Holdsworth, 2014; Gibbs, Isa, et al., 2014; Varshavsky, 2019). The pathways were originally identified in *Saccharomyces cerevisiae* (Bachmair, Finley, & Varshavsky, 1986) and have been shown to occur in all branches of life examined. The eukaryotic branches originally identified in yeast and mammalian systems have been shown to function in plants, principally by analysis of the stabilities of Nt residues and by assessing the activities of enzymic components in vitro, in plants, or in yeast (Graciet, Mesiti, & Wellmer, 2010; Graciet et al., 2009; Millar et al., 2019; Mot et al., 2018) (Figure 1a). A final stage of the pathways is recognition of destabilizing Nt residues of substrates by N‐recognin E3 ligases and subsequent degradation via the ubiquitin proteasome system (UPS). In mammals and yeast, N‐recognin E3 ligases are represented by proteins that recognize both type 1 (basic) and type 2 (hydrophobic bulky) Nt residues. *Saccharomyces cerevisiae* has a single N‐recognin, Ubr1p, whereas mammals have at least four (UBR1, 2, 4, and 5), belonging to three different protein families (Varshavsky, 2019). These proteins contain two separate domains that bind either type 1 or type 2 destabilizing residues. In *Arabidopsis thaliana* (hereafter arabidopsis), two structurally distinct N‐recognins recognize either type 1 (PROTEOLYSIS[PRT]6) or aromatic type 2 (PRT1) residues. PRT6 was originally identified via its sequence similarity to Ubr1p and its amino acid specificity confirmed using model reporters (Garzon et al., 2007). The first clues to its physiological roles were obtained through a forward genetic screen to define regulators of seed germination (Holman et al., 2009). PRT1 was cloned following a genetic screen that identified components required for destabilization of an artificial substrate initiating with phenylalanine (F‐dihydrofolate reductase; F‐DHFR) and was shown to encode a protein with a novel structure consisting of two RING finger domains and one ZZ domain, with specificity for aromatic amino‐terminal residues (Bachmair, Becker, & Schell, 1993; Potuschak et al., 1998; Stary et al., 2003). It is unclear why recognition determinants of type 1 and 2 residues are encoded by two unrelated proteins, or why type 2 residue recognition was lost from UBR‐like proteins in plants.

Very little is known regarding the physiological functions of the PRT1 N‐degron pathway, and only a single report investigated a role in plant defense (de Marchi et al., 2016). One substrate was identified for PRT1, the E3 ligase BIG BROTHER (Dong et al., 2017). In contrast, the PRT6 pathway has been shown to influence many stages of plant growth and development, and interactions with the environment. The Cys branch of the PRT6 N‐degron pathway acts as a sensor for oxygen and nitric oxide (NO), controlling tolerance to abiotic stresses including hypoxia, drought, salinity and heat shock, and also regulates protease activities and storage reserve mobilization (Abbas et al., 2015; Gibbs et al., 2015, 2011; Gibbs, Bacardit, et al., 2014; Gibbs, Isa, et al., 2014; Licausi et al., 2011; Mendiondo et al., 2016; Vicente et al., 2017; Zhang, Gannon, Hassall, et al., 2018; Zhang, Gannon, Jones, et al., 2018). The NTAQ1 (Nt‐Gln amidohydrolase) branch of the same pathway influences the plant immune response by controlling synthesis of the major arabidopsis phytoalexin camalexin and by influencing expression of pathogenesis‐associated proteins (Vicente et al., 2019).

Protease activity is a key regulator of protein abundance and function during the plant immune response, as peptide cleavage, which may reveal a new Nt‐destabilizing residue, can be a mechanism to remove activity of the target or may result in a new activity for the polypeptide product (Tuominen et al., 2019; Zhou & Zeng, 2017). Plants can recognize pathogens at the site of infection by different layers of defense. PAMP‐triggered immunity (PTI) is induced upon recognition of conserved pathogen molecules, such as lipopolysaccharides or flagellin, called pathogen‐associated molecular patterns (PAMPs) (Boller & Felix, 2009). Effector‐triggered immunity (ETI) is activated when effector proteins delivered into the plant cell by the pathogens are recognized by plant resistance (R) gene products. Onset of ETI also primes distal uninfected tissues against a potential secondary infection by inducing systemic acquired resistance (SAR) (Durrant & Dong, 2004; Grant & Lamb, 2006). The response initiated in the infected tissue is transmitted to distal uninfected tissues by linear or parallel activity of several molecules: methyl salicylate (MeSA), azelaic acid (AzA), glycerol 3‐phosphate (G3P), NO, reactive oxygen species (ROS), galactolipids (GA), and pipecolic acid (Pip) (Wang et al., 2018).

In this paper, we report the effects of loss of PRT1 function on the immune system. A previous report showed that the *prt1* mutant was more susceptible to the hemi‐biotroph bacterium *Pseudomonas syringae* pv *tomato* in short‐day plants (de Marchi et al., 2016); however, using an assembly of different approaches, we show that removal of the activity of the PRT1 E3 ligase enhances resistance of arabidopsis to this pathogen, and that this is associated with constitutive alteration of the proteome including the enhanced accumulation of many pathogenesis‐associated proteins. In addition, we show an altered metabolome profile in the mutant including SA, camalexin, and, together with *prt6* mutant, Pip.

## 2 MATERIALS AND METHODS

### 2.1 Plant material, growth conditions, and experimental design

*Arabidopsis thaliana* seeds were obtained from Nottingham Arabidopsis Stock Centre (NASC), UK, unless otherwise stated, including *prt1‐1* (N119, *Q111‐STOP) and *prt6‐1* (SAIL 1278_H11). The *prt1 prt6* double mutant (Garzon et al., 2007) was the kind gift from Andreas Bachmair (University of Vienna). All mutants are in the Col‐0 (wild type, WT) accession. Plants were grown and assays performed in controlled‐environment rooms under the following conditions: 12 hr of light (23°C) and 12 hr of dark (18°C), 60%–70% relative humidity, under white fluorescent light (120 mmol/m^2^ s^−1^). Plants were treated between 3 and 4 weeks after germination.

### 2.2 Construction of the PRT1 complementing line

The 1.1‐kb promoter of *PRT1* was amplified from genomic DNA with the primer pair AttB4_PRT1pro_For and AttB1R_PRT1pro_Rev and the full‐length genomic sequence of *PRT1* without stop codon was amplified with primer pairs AttB1_PRT1_For and AttB2_PRT1_nonstop. These products were then recombined into pDONR P4‐P1R and pDONR221 vectors (Invitrogen) to generate pEN‐L4‐promPRT1‐R1 and pEN‐L1‐gPRT1‐L2, respectively. Both of the entry vectors were sequenced, and recombination reactions were carried out with pEN‐R2‐GStag‐L3 and pKCTAP (Van Leene et al., 2008) to generate construct MO14, using a Multisite Gateway^®^ LR Recombination Reaction (Invitrogen), according to manufacturer's instructions. Transformation into *Agrobacterium tumefaciens* (strain AGL‐1) and *Arabidopsis thaliana* *prt1‐1 *were performed according to established protocols (Clough & Bent, 1998), and transgenic plants were selected using kanamycin and subsequently methotrexate.

### 2.3 Analysis of pathogen growth in plant material

The bacterial suspension was injected with a needleless syringe into the abaxial side of leaves or sprayed on the surface of the leaves of 3.5‐week‐old plants. *Pst* DC3000 *avrRpm1* and *Pst* DC3000 were grown overnight at 28°C in Petri plates with King's B medium. For analysis of bacterial growth, three leaves of intermediate age per plant of at least seven plants were injected with a bacterial suspension of 10^6^ cfu/ml (O.D_600nm_ 0.1 = 10^8^ cfu/ml) or sprayed with a suspension of 10^8^ cfu/ml. For analyses of systemic acquired resistance, three leaves of at least seven plants were pre‐treated with a bacterial suspension of *Pst* DC3000 *avrRpm1* of 10^8^ cfu/ml and, 48 hr later, three upper leaves were subjected to a second bacterial inoculation of *Pst* DC3000 of 10^6^ cfu/ml. A disk of 0.28 cm^2^ from each infected leaf was excised at 96 hr, pooled in triplicate, homogenized, diluted, and plated for counting.

### 2.4 Measurement of ion leakage

Cell damage was determined by measuring ion leakage as described (de Leon, Sanz, Hamberg, & Castresana, 2002). 25‐days‐old Arabidopsis were injected with the bacterial suspension of 10^8^ cfu/ml. Three leaves per plant of eight plants were treated. A disk of 0.6 cm^2^ per leaf from 24 leaves was excised using a hole punch, then rinsed briefly with water, and floated on 5 ml of double‐distilled water for 6 hr at room temperature. The conductivity of the water was measured using a Mettler‐Toledo SevenGo conductivity meter.

### 2.5 Proteomic analysis

Proteins were extracted from three leaves 4 hr after lights on from 28‐days‐old Col‐0 and *prt1‐1* plants. Protein extraction, quantification, reduction, and alkalization were done as in Zhang et al. (2015). Protein precipitation was done by methanol/chloroform method as in Zhang, Gannon, Hassall, et al. (2018) and sequential trypsin digestion performed according to Zhang et al. (2015). Peptide concentration was determined using a Pierce^TM^ Quantitative Colorimetric Peptide Assay kit (23,275, Thermo Scientific). 100 µg peptide aliquots were labeled using TMT10plex^TM^ (90,110, Thermo Scientific). The experimental design included five biological replicates: Col‐0 was labeled with TMT10 −126, −127N, −127C, −128N, −128C, and *prt1‐1* was labeled with −129N, −129C, −130N, −130C, −131. Equal amounts of peptides were mixed and peptides from half of this mixture were separated by high pH reverse‐phase chromatography using a Waters reverse‐phase nano column as described in Zhang et al. (2015). LC‐MS/MS was performed as previously (Zhang, Gannon, Hassall, et al., 2018; Zhang, Gannon, Jones, et al., 2018) using a Dionex Ultimate 3,000 RSLC nanoUPLC (Thermo Fisher Scientific Inc, Waltham, MA, USA) system and a Orbitrap Fusion Lumos Tribrid Mass Spectrometer (Thermo Fisher Scientific Inc, Waltham, MA, USA). Raw data were searched against the TAIR10 database using MASCOT v.2.4 (Matrix Science, London, UK) and PROTEOME DISCOVERER™ v.1.4.1.14, employing Top 10 peaks filter node and percolator nodes and reporter ions quantifier with trypsin enzyme specificity with a maximum of one missed cleavage. Carbamidomethylation (+57.021 Da) of cysteine and TMT isobaric labeling (+229.162 Da) of lysine and N termini were set as static modifications while the methionine oxidation (+15.996) were considered dynamic. Mass tolerances were set to 20 ppm for MS and 0.6 Da for MS/MS. For quantification, integration tolerance was set to 2mmu. Purity correction factor was set according to the TMT10plex (90,110) product sheet (Lot number: SA239883). Each reporting ion was divided by the sum of total ions and normalized by medians of the samples. Overall p‐values were obtained using two‐sample *t* test of log_2_‐transformed data, considering variation of quantification as a weighting factor. Gene ontology analysis was performed using Panther14.1 (http://www.pantherdb.org).

### 2.6 Protein extraction and Immunoblotting

Protein extraction and immunoblotting were carried out as described in Zhang, Gannon, Hassall, et al. (2018), Zhang, Gannon, Jones, et al. (2018), using the following antisera: rabbit anti‐PR2 (Agrisera; AS12 2,366; 1:1,000 dilution) or mouse anti‐SBP (SB19‐C4, Santa Cruz; 1:300 dilution). PR2 and SBP blots were developed using SuperSignal™ West Pico PLUS Chemiluminescent Substrate and SuperSignal™ West Femto Maximum Sensitivity Substrate (ThermoFisher Scientific), respectively.

### 2.7 Gene expression analysis

RNA extraction, cDNA synthesis, quantitative RT–PCR were performed as previously described (Gibbs, Bacardit, et al., 2014; Gibbs, Isa, et al., 2014; Gibbs et al., 2011). For primers used, see Table S2.

### 2.8 Metabolome analysis, liquid chromatography, and mass spectrometry for targeted metabolomics

Fresh material was harvested, powdered, and stored at −80°C until freeze‐dried. The procedure for hormone analysis was described previously (Sánchez‐Bel et al., 2018). Before extraction, 30 mg of lyophilized material was supplemented with 100 ng/ml of internal standards (ABA‐d_6_, SA‐d_5_, dhJA, and JA‐Ile‐d_6_). The aqueous extraction was set at pH 2.6 with acetic acid 30% and partitioned twice with diethyl ether. After evaporation, the pellet was dissolved in water/methanol (9:1). The final solution was filtered over a 0.22‐µm cellulose filter (RC membrane filter, 15 mm, Phenomenex). The chromatographic separation was conducted in a UPLC analytical column Kinetex EVO C18, 2.6 µm particle size, 2.1 × 50 mm (Phenomenex). Chromatographic and mass spectrometry analysis was performed after injection of an aliquot of 20 µl into the Acquity Ultra‐Performance Liquid Chromatography system (UHPLC; Waters, Mildford, MA, USA) coupled to a triple quadrupole mass spectrometer. All the parameters and conditions for chromatography and mass analyzer was published previously (Gamir, Pastor, Cerezo, & Flors, 2012). Pipecolic acid was extracted with 1 ml solution of 0.1% of HCOOH in 30mg of lyophilized plant material and supplemented with 100 ng/ml of Phe‐13C_9_ 15N as internal standard. The extraction was performed in a mixer mill with frequency of 30Hz, 3 min. After centrifugation and filtration, samples were diluted 5 times with 0.1% of HCOOH, and 10 µl of the dilution was injected on an Acquity UPLC column HSS T3 2.1 x 100 mm, 1.8 µm, maintained at 40°C, in the same instrument as mentioned above. For chromatographic elution of the analyte, water and methanol were used as mobile phases, with no additional additives. The elution was carried out with a flux of 0.3 ml/min solvent elution, starting with 95% of aqueous mobile phase and kept in isocratic conditions along 2 min, reaching 10% of the aqueous mobile solvent at 3 min and let to recover the initial conditions at 6 min. The column was allowed to equilibrate for 4 min, giving a total of 10 min per sample. Ion detection was set in positive electrospray ionization and all the mass analyzer conditions were the same as described for hormone analysis. The cone and collision energies were 20 V and 25 eV for pipecolic acid, and 30 V and 15 eV, respectively, for Phe‐13C_9_ 15N. The transition selected for pipecolic acid was 130.1 > 56.1, and for the internal standard 175.1 > 128.

### 2.9 Experimental statistical analyses

Experiments were performed at least in triplicate. Horizontal lines represent standard error of the mean values in all graphs. Student's *t* test significant differences are reported as ∗∗∗ (*p* < .001), ∗∗ (*p* < .01), ∗ (*p* < .05), one‐way ANOVA (analysis of variance) with Tukey's multiple comparisons test, where significant differences (alpha < 0.05) are denoted with different letters (GraphPad Prism 7.0 software).

## 3 RESULTS

### 3.1 The evolution of N‐recognins in plants

In arabidopsis, PRT6 and PRT1 provide complementary N‐recognin functions for type 1 and 2 Nt residues, respectively (Figure 1a). Previous analysis showed that PRT6 does not contain the ClpS‐like domain required for recognition of N‐degrons containing type 2 residues and is therefore only able to recognize basic destabilizing residues (Garzon et al., 2007). Investigation of the presence of this domain in PRT6‐like sequences from different taxonomic groups showed that, in addition to its absence in flowering plants, it is also absent from other plant and plant‐like taxa, including from species within algae and algae‐like taxa (Fig. S1), indicating an early loss of the domain during the evolution of plants. Analysis of PRT1‐like sequences, containing two Zn‐RING domains and a ZZ domain, demonstrated their presence and conserved organization within proteins in all sequenced land plant groups, and taxa represented in charophyte and chlorophyte algae but not taxa outside these domains (Figure 1b; Fig. S1). This demonstrates an early separation of the known N‐recognin specificities in the evolution of plants.

### 3.2 PRT1 modulates resistance against Pst DC3000

Our analysis of PRT1 function was carried out using a mutant allele of *PRT1* (AT3G24800), *prt1‐1*, where a C to T transition generates a premature stop codon (Q111*) (Potuschak et al., 1998) (Fig. S2). This mutant displays a rosette with no observable external phenotypic differences from wild type (Fig. S2). As no loss‐of‐function T‐DNA insertion mutants exist for this gene, a transgenic line was generated in which a transgene consisting of the *PRT1* genomic region fused to the GS‐Tag (Van Leene, Witters, Inze, & Jaeger, 2008) driven by its 1.1 kb promoter was introduced into the *prt1‐1* mutant. This construct provided complete complementation of the *prt1‐1* methotrexate‐resistant F‐DHFR stabilizing phenotype (Fig. S2). The PRT1‐tag protein was detected in roots but not in shoots of 7‐day‐old plants and was stabilized by application of proteasome inhibitor in both organs, indicating that PRT1 itself is turned over by the UPS, in common with many E3 ligases (de Bie & Ciechanover, 2011). PRT1‐tag was also undetectable in leaves of 28‐day‐old plants (Fig. S2).

**Figure 2:**
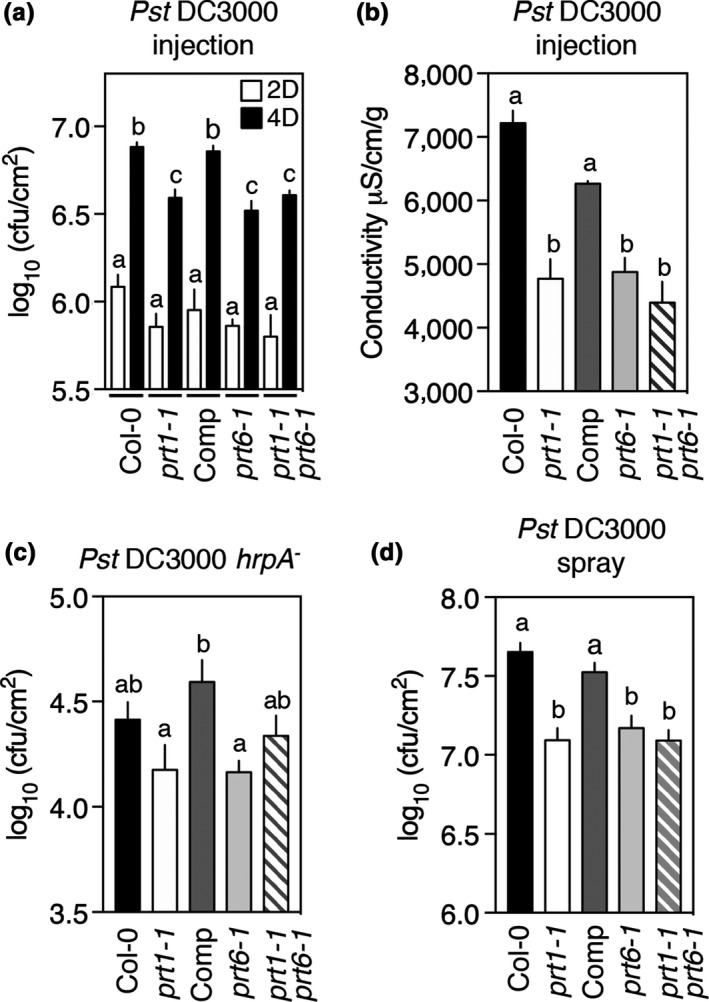
*prt1‐1* mutation alters plant response to *Pst* DC3000. (a) Quantification of *Pst* DC3000 growth in WT and mutant plants 2 and 4 days after bacterial infiltration by injection (10^6^ cfu/ml). (b) Ion leakage measurement in leaves 4 days after infiltration with *Pst* DC3000 (10^7^ cfu/ml). (c) Quantification of Pst DC3000 *hrpA^‐^* (10^6^ cfu/ml) growth 4 days after injection. (d) Quantification of *Pst* DC3000 growth in WT and mutant plants 4 days after bacterial foliar spray application (10^8^ cfu/ml). Data represent means ± *SEM*. Statistical differences were analyzed by ANOVA followed by Tukey test (*p* < .05) or Student's *t* test **p < .01

In order to investigate a possible role for PRT1 in the plant immune system, the function of PRT1 in modulating response to the model bacterial pathogen *Pseudomonas syringae* pv *tomato* DC3000 (hereafter *Pst* DC3000) was evaluated. Following infection with *Pst* DC3000, bacterial growth in leaves of *prt1‐1* was significantly lower than in Col‐0 (wild‐type, WT) or the complemented line 4 days post‐infiltration, whereas the complemented *prt1‐1* line showed a response similar to WT (Figure 2a). The *prt1‐1* mutant exhibited enhanced resistance to *Pst* DC3000 infection, similar to that shown by *prt6‐1*, that we previously demonstrated (Vicente et al., 2019). A double mutant, *prt1 prt6*, removing both PRT1 and PRT6 N‐recognin activities was generated to assess a possible combined effect. The double mutant also showed significantly lower bacterial growth than the WT, similar to both single mutants *prt1‐1* and *prt6‐1* (Figure 2a). The reduction in bacterial growth, and therefore cell damage, correlates with tissue cellular‐leakage measured 4 days following infection that was significantly lower in *prt1‐1*, *prt6‐1,* and the double mutant but not different between WT and the *prt1‐1* complemented line (Figure 2b). These results indicate that disruption of PRT1 activity enhances plant defense against *Pst* DC3000, similar to what was described previously for PRT6 (Vicente et al., 2019). Inoculation with the PTI inducer *Pst* DC3000 *hrpA^‐^* (with a compromised type‐three secretion system) did not show significant differences in bacterial growth between WT and any of the mutants (Figure 2c). Both *prt1‐1* and *prt1 prt6* exhibited increased resistance when *Pst* DC3000 was applied to leaves by foliar spray application (Figure 2d).

### 3.3 PRT1 works separately to PRT6 to influence resistance to Pst DC3000

**Figure 3:**
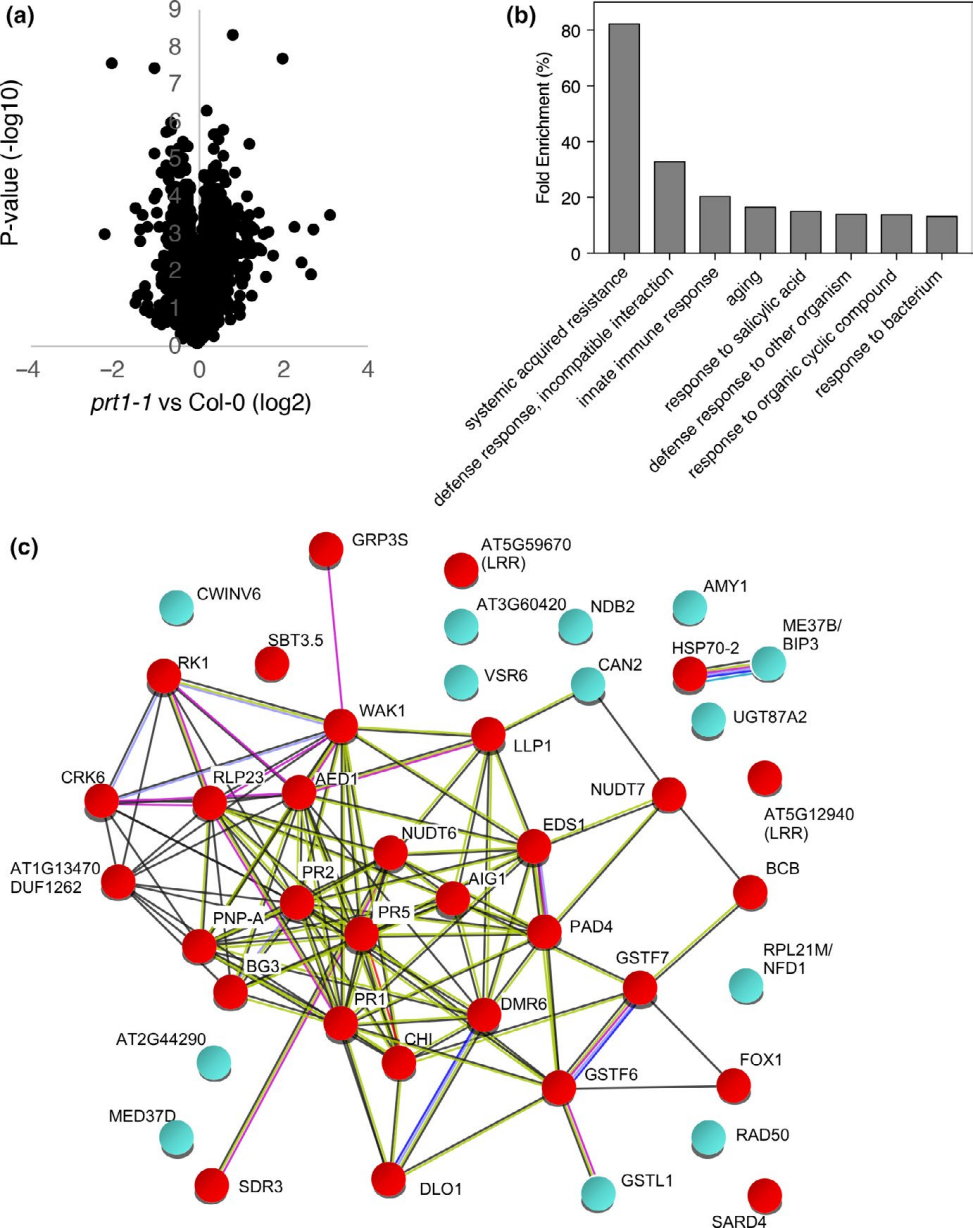
Analysis of proteins differentially regulated in untreated *prt1‐1* leaves. (a) Analysis of protein quantification in Col‐0 and *prt1‐1.* Volcano plot showing the relationship between statistical significance (*p*‐value) on the y‐axis and the biological significance (log2 fold change) on the x‐axis. (b) Gene ontology analysis of proteins upregulated in untreated *prt1‐1*, showing the fold enrichment compared with their normal appearance in WT plants. (c) Network diagram indicating relationships of proteins upregulated in *prt1‐1* (defined as proteins with ≥2‐fold change in abundance at *p* ≤ .05, which are represented by at least two unique peptides). Figure was generated using String (Szklarczyk et al., 2019) and edited to indicate proteins (nodes) associated with defense (red), defined by the Analysis function of String and by examination of the literature. Edges represent interactions, color‐coded as follows: cyan, from curated databases; magenta, experimentally determined; green, gene neighborhood; red, gene fusions; blue, gene colocalization; lime, text mining; black, co‐expression; lilac, protein homology

In the *prt1‐1* mutant, substrates with type 2 Nt‐destabilizing residues are constitutively stable in untreated plants (Bachmair et al., 1993; Potuschak et al., 1998; Stary et al., 2003) and could therefore contribute to the observed increased resistance to *Pst* DC3000 by enhancing the defense response. Therefore, to uncover a molecular role of PRT1 in the immune response, we conducted a quantitative proteomic analysis incorporating tandem mass tag (TMT™) labeling to compare proteomes of untreated WT and *prt1‐1* adult leaves. 7,398 proteins were quantified in untreated leaves of Col‐0 and *prt1‐1* plants, and PRT1, represented by a single peptide, was identified as the most downregulated protein (Table S1). 5,541 proteins were represented by at least two unique peptides (Figure 3; Table S1). Of these, 55 were differentially regulated in *prt1‐1* (defined by a ≥2‐fold change in abundance at *p* ≤ .05), 44 of which exhibited increased abundance in *prt1‐1*, and 11 decreased (Table 1). Enrichment analysis revealed that a large group of the upregulated proteins has a role in plant response to stress, specifically defense against pathogenic infections (Figure 3b,c). Further analysis revealed that the observed increased protein levels were accompanied by transcript accumulation (Fig. S3), which was enhanced in the double‐mutant *prt1 prt6*. This suggests that PRT1 (and PRT6) substrates responsible for increased *Pst* resistance function upstream of the transcription of the genes that code for these proteins. To examine further the relationship between transcriptional regulation and proteins with altered abundance in *prt1‐1*, the Signature tool of GENEVESTIGATOR (Zimmermann, Hirsch‐Hoffmann, Hennig, & Gruissem, 2004) was used to identify transcriptome datasets with similar patterns of regulation to the *prt1‐1* proteome (Fig. S4). The proteome signature of *prt1‐1* leaves resembled transcriptome signatures of pathogen‐treated plants, of mutants and transgenics which lack or over‐express key regulators of immunity and of plants in which SA was perturbed genetically or by exogenous application.

**Table 1:** Proteins with altered abundance in uninfected *prt1‐1* leaves

Accession	Description	Synonyms	Ratio	P value	Score	Cov.
AT3G57260.1	Beta‐1,3‐glucanase 2	PR‐2	8.80	0.0004104	408.91	28.61
AT1G09080.1	Heat‐shock protein 70 (Hsp 70) family protein	MED37B; BIP3	6.63	0.0008866	168.03	10.96
AT2G43570.1	Chitinase, putative	CHI; AED15	6.42	0.0144008	165.56	28.52
AT1G75040.1	Pathogenesis‐related gene 5	PR‐5	5.60	0.0071701	377.38	56.07
AT5G10760.1	Eukaryotic aspartyl protease family protein	AED1	4.96	0.0007452	101.34	5.6
AT5G59670.1	Leucine‐rich repeat protein kinase family protein		4.05	2.419E‐08	67.26	2.3
AT3G57240.1	Beta‐1,3‐glucanase 3	BG3; GNS3	3.42	0.0045561	303.77	29.03
AT1G02920.1	Glutathione S‐transferase 7	GST7; GSTF6	3.24	0.00115	311.66	51.67
AT2G14610.1	Pathogenesis‐related gene 1	PR‐1	3.06	0.0182382	189.57	22.98
AT2G04450.1	Nudix hydrolase homolog 6	NUDX6; NUDT6	3.02	0.0011908	185.22	28.27
AT2G44290.1	Bifunctional inhibitor/lipid‐transfer protein/seed storage 2S albumin superfamily protein		2.81	0.0034306	61.37	9.27
AT5G20230.1	Blue‐copper‐binding protein	BCB; SAG14	2.79	0.0029616	118.54	14.8
AT2G18660.1	Plant natriuretic peptide A	PNP‐A; ECG2	2.75	0.0003511	97.29	30.77
AT5G18470.1	Curculin‐like (mannose‐binding) lectin family protein	TCAN2; CAN2	2.63	0.0030977	114.54	12.35
AT4G23140.2	Cysteine‐rich RLK (RECEPTOR‐like protein kinase) 6	CRK6	2.54	0.0009568	40.67	5.29
AT5G52810.1	NAD(P)‐binding Rossmann‐fold superfamily protein	SARD4	2.46	0.0037914	97.13	19.38
AT2G30140.1	UDP–glycosyltransferase superfamily protein	UGT87A2	2.40	0.0035203	140.98	9.45
AT2G31970.1	DNA repair‐recombination protein (RAD50)	AtRAD50	2.39	0.0008481	27.50	1.52
AT5G12940.1	Leucine‐rich repeat (LRR) family protein		2.38	4.857E‐06	108.98	10.24
AT2G32680.1	Receptor‐like protein 23	RLP23	2.37	0.0022937	89.88	10.11
AT4G30930.1	Ribosomal protein L21	RPL21M; NFD1	2.36	0.001087	78.78	8.52
AT1G30900.1	VACUOLAR SORTING RECEPTOR 6	VSR6; VSR3;3; BP80‐3:3	2.33	0.0023571	199.97	13.63
AT1G21250.1	Cell wall‐associated kinase	WAK1; PRO25	2.31	0.0003757	349.48	14.42
AT4G32610.1	Copper ion binding	MED37D	2.30	0.0014406	84.36	11.75
AT5G02490.1	Heat‐shock protein 70 (Hsp 70) family protein	HSP70‐2	2.30	0.0038743	1,082.85	41.35
AT4G12720.4	MutT/nudix family protein	AtNUDT7	2.29	0.0001146	116.48	16.77
AT3G60420.2	Phosphoglycerate mutase family protein		2.26	0.0006045	89.05	9.43
AT1G32940.1	Subtilase family protein	SBT3.5	2.25	0.0089578	104.97	4.91
AT3G52430.1	Alpha/beta‐hydrolases superfamily protein	PAD4	2.25	0.0008327	99.04	9.98
AT1G02930.1	Glutathione S‐transferase 6	GST6	2.23	0.0086252	311.64	47.12
AT1G13470.1	Protein of unknown function (DUF1262)		2.20	0.0179727	48.53	6.07
AT2G05380.1	Glycine‐rich protein 3 short isoform	GRP3S	2.19	0.0173964	468.58	43.1
AT5G02780.1	Glutathione transferase lambda 1	GSTL1	2.19	0.0161203	49.89	10.97
AT4G25000.1	Alpha‐amylase‐like	AMY1	2.18	0.0011308	101.35	7.8
AT1G26380.1	FAD‐binding Berberine family protein	FOX1	2.12	0.012175	64.86	9.16
AT1G33960.1	P‐loop containing nucleoside triphosphate hydrolases superfamily protein	AIG1	2.08	0.017869	120.12	23.51
AT4G10500.1	2‐Oxoglutarate (2OG) and Fe(II)‐dependent oxygenase superfamily protein	DLO1	2.06	0.0026657	99.94	14.9
AT2G47130.1	NAD(P)‐binding Rossmann‐fold superfamily protein	SDR3	2.05	0.0260519	174.09	25.68
AT3G48090.1	Alpha/beta‐hydrolases superfamily protein	EDS1	2.04	0.0008192	140.47	18.46
AT5G03350.1	Legume lectin family protein	LLP1; AED9	2.02	0.0003634	250.78	36.5
AT4G05020.2	NAD(P)H dehydrogenase B2	NDB2	2.02	0.0135213	268.21	18.42
AT5G24530.1	2‐oxoglutarate (2OG) and Fe(II)‐dependent oxygenase superfamily protein	DMR6	2.02	0.0130101	244.18	21.99
AT1G65790.1	receptor kinase 1	RK1; SD17	2.01	0.004197	98.29	4.03
AT5G11920.1	6‐&1‐Fructan exohydrolase	CWINV6	2.01	0.0122742	82.80	13.64
AT5G20250.4	Raffinose synthase family protein	DIN10; RS6; RFS6	0.50	8.896E‐06	118.28	8.53
AT1G22530.1	PATELLIN 2	PATL2	0.49	4.559E‐08	1,082.92	56.66
AT4G09160.1	SEC14 cytosolic factor family protein/ phosphoglyceride transfer family protein	PATL5	0.43	0.0008063	30.90	6.59
AT5G05890.1	UDP–glycosyltransferase superfamily protein	UGT76C5	0.42	0.0289026	96.00	5.27
AT4G30270.1	Xyloglucan endotransglucosylase/hydrolase 24	XTH24; MERI‐5; SEN4; MERI5B	0.41	0.0497484	121.88	13.75
AT3G25760.1	Allene oxide cyclase 1	AOC1; ERD12	0.41	0.000368	175.94	19.29
AT3G16420.1	PYK10‐binding protein 1	PBP1	0.39	0.0009317	185.95	21.81
AT2G23120.1	Late embryogenesis abundant protein, group 6		0.39	0.0019211	95.59	81.93
AT3G15950.1	DNA topoisomerase‐related	NAI2	0.36	0.0002488	205.76	16.19
AT5G61160.1	Anthocyanin 5‐aromatic acyltransferase 1	AACT1; ACT	0.24	3.192E‐08	62.21	5.75
AT2G45180.1	Bifunctional inhibitor/lipid‐transfer protein/seed storage 2S albumin superfamily protein		0.22	0.0013174	43.05	11.19

Note

Table shows the normalized ratios of protein abundance, determined by TMT™ labeling of proteins extracted from 28‐days‐old uninfected leaves (average of five biological replicates). Increased abundance was defined as a twofold change in *prt1‐1*, relative to Col‐0. Only proteins represented by ≥2 peptides are shown; all proteins identified and quantified are presented in Table S1. Cov, Indicates percent coverage.

**Figure 4:**
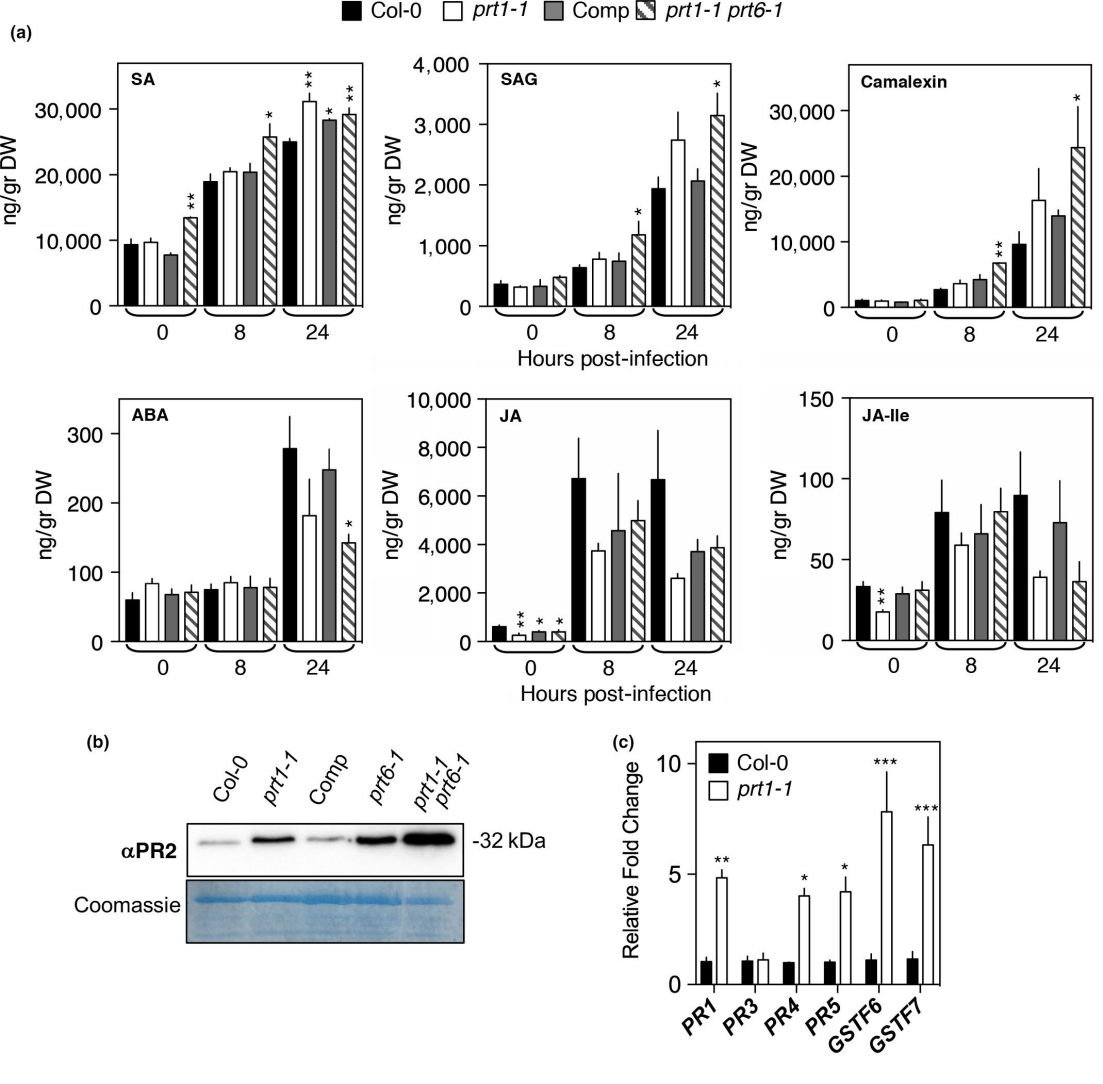
Changes in defense phytohormone, protein, and transcript levels in WT *prt1,* complementing line *PRT1*/*prt1‐1, prt1prt6* with or without *Pst* DC3000 infection. (a) Quantification of phytohormones in mature plants infiltrated with or without *Pst* DC3000 (10^7^ cfu/ml). SA, salicylic acid; SAG, salicylic acid beta‐glucoside; ABA, abscisic acid; JA, jasmonic acid; JA‐Ile, jasmonic acid isoleucine conjugate. (b) Accumulation of PR2 protein in untreated mature plants. CBB, Coomassie Brilliant Blue. (c) Relative expression of transcripts encoding defense and stress‐related genes in untreated WT and *prt1‐1* plants. Data represent means ± *SEM*. Statistical differences were analyzed by Student's *t* test *p < .05, **p < .01, *p < .05, **p < .01, ***p < .001

Within the group of proteins whose accumulation was enhanced in *prt1‐1* were several key pathogenesis‐related proteins, including PATHOGENESIS RELATED (PR) PR1 (AT2G14610; ratio 3.06), an extracellular protein instrumental for resistance against *Pst* DC3000 infections, and ENHANCED DISEASE SUSCEPTIBILITY (EDS)1 (AT3G48090; ratio 2.04) and PHYTOALEXIN DEFICIENT(PAD)4 (AT3G52430; ratio 2.25), two lipase‐like proteins that, associated or individually, positively regulate basal and effector‐triggered defense responses (Feys, Moisan, Newman, & Parker, 2001; Wiermer, Feys, & Parker, 2005; Zhou, Tootle, Tsui, Klessig, & Glazebrook, 1998). In addition, six apoplastic EDS1‐DEPENDENT (AED) proteins (Breitenbach et al., 2014) were also upregulated in untreated *prt1‐1*: PR2 (AT3G57260; ratio 8.80), PR5 (AT1G75040; ratio 5.6), PLANT NATRIURETIC PEPTIDE A (PNP‐A) (AT2G18660; ratio 2.75), recently described as a tissue damage protector during pathogenic infections (Ficarra et al., 2017), a PUTATIVE CHITINASE (AT2G43570; ratio 6.42), and, interestingly, a LECTIN FAMILY PROTEIN (LLP1) (AT5G03350 ratio; 2.02) and an ASPARTYL PROTEASE (AED1) (AT5G10760 ratio; 4.96), described as essential positive regulators and suppressors of SAR, respectively (Breitenbach et al., 2014). It has been suggested that AED1 may degrade apoplastic proteins, including PRs, accumulated during this systemic response as a feedback mechanism. The fact that EDS1 solely controls the accumulation of a pool of proteins with antagonistic roles illustrates the need for a homeostatic mechanism to control the extent of SAR. Although signaling from the EDS1/PAD4 hub can work independently of SA, (Cui et al., 2017), its role in promoting SA‐mediated basal resistance has been extensively reported (Rietz et al., 2011). In line with the observed upregulation of SA‐associated proteins, *prt1‐1* leaves infected with *Pst* DC3000 showed slightly increased levels of SA compared to WT (Figure 4a). The *prt1 prt6* double mutant showed increased SA levels even in absence of the pathogen that, together with SAG, was higher than *prt1‐1* and WT during the time course after pathogen inoculation. These results indicate that resistance displayed by these mutants would be, at least partially, SA dependent, and are consistent with previous data showing that increased resistance to *Pst* DC3000 in *prt6* mutants is dependent on SID2, an enzyme involved in SA synthesis (Vicente et al., 2019).

It has been shown previously that mutating PAD3, the enzyme that catalyzes the final steps of the synthesis of the phytoalexin camalexin, reverts the enhanced resistance of *prt6* (Vicente et al., 2019). In a similar way, proteomic analysis of *prt1‐1* revealed an increased accumulation of GLUTATHIONE S‐TRANSFERASE (GST)F6 and GSTF7 (AT1G02930.1; ratio 2.23 and AT1G02920; ratio 3.24), that have an established role and a putative role, respectively, in the synthesis of camalexin (Su et al., 2011). Although *prt1‐1* exhibited a slight increase in the levels of camalexin during *Pst* DC3000 infections (Figure 4a), levels were significantly greater in *prt1 prt6*. *prt1‐1* plants also showed increased abundance of FAD‐binding Berberine family protein FOX1 (AT1G26380; ratio 2.12), involved in the synthesis of the phytoalexin 4‐hydroxyindole‐3‐carbonyl nitrile (4‐OH‐ICN), a cyanogenic compound involved in inducible pathogen defense (Rajniak, Barco, Clay, & Sattely, 2015). Camalexin and 4‐OH‐ICN are both synthesized from a common precursor, indole‐3‐acetaldoxime (IAOx), that together with the differential accumulation of camalexin in *prt1‐1* compared to *prt1‐1 prt6‐1* indicates that PRT1 and NTAQ1/PRT6 activities independently contribute to the synthesis of phytoalexins. An increased abundance of the defense‐associated protein PR2 (AT3G57260.1; ratio 8.80) detected in *prt1‐1* was also observed in *prt6‐1*, and a greater accumulation observed in the double *prt1 prt6* (Figure 4b). In line with this, an increased expression of SA‐responsive *PR1* and *PR5* and *GSTF6/7* genes was also detected in uninfected plants (Figure 4c). Surprisingly, we also observed an increase in the JA and ET responsive *PR4,* although the proteomics data indicate no protein abundance change (AT3G04720.1; ratio 0.90).

Jasmonic acid (JA), and its active form JA‐Ile, induce susceptibility to *Pst* DC3000 by interfering with SA‐mediated signaling pathways (Pieterse, Van der Does, Zamioudis, Leon‐Reyes, & Van Wees, 2012). Among the proteins downregulated in *prt1‐1*, two are associated with biosynthesis and response to JA, respectively, ALLENE OXIDE CYCLASE 1 (AOS1) (AT3G25760.1 ratio 0.41) was significantly reduced and a single peptide of PLANT DEFENSIN 1.3 (PDF1.3) (AT2G26010.1; ratio 0.08) was observed. Slightly lower levels of JA and JA‐Ile were observed in both *prt1‐1* and *prt1‐1 prt6‐1* compared with WT (Figure 4a). In addition, it has been demonstrated recently that EDS1/PAD4 interferes during ETI with MYC2, a master regulator of JA signaling pathway (Cui et al., 2018). No statistically significant differences in OPDA levels were observed in untreated or infected tissue of mutants or WT (Fig. S5). Abscisic acid (ABA) plays a positive role during pre‐invasive penetration resistance, specifically controlling stomatal movement, and a negative role on later post‐invasive events by interfering with callose deposition or SA synthesis (Ton, Flors, & Mauch‐Mani, 2009). In line with the observed increased resistance against *Pst* DC3000, *prt1 prt6* levels contained lower levels of ABA compared with WT (Figure 4a). Together, these data clearly indicate that disruption of PRT1 activity influences plant immune responses by regulating the accumulation of a significant pool of defense proteins and that this role is complementary to that of the known roles of PRT6 (Vicente et al., 2019).

### 3.4 Removal of PRT1 activity primes plants against Pst DC3000 infections

**Figure 5:**
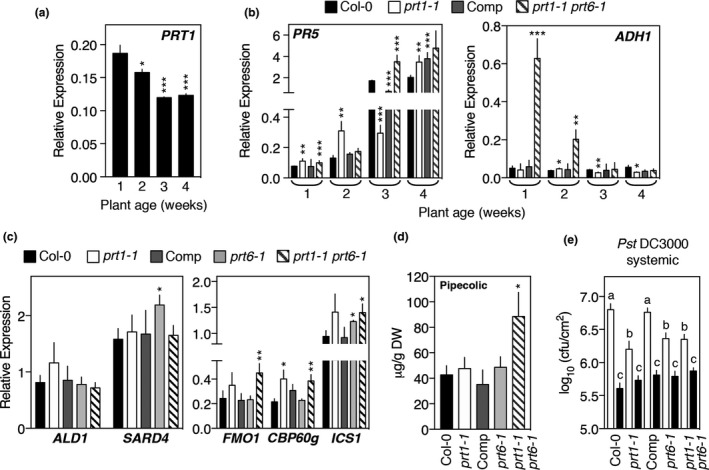
*prt1‐1* mutation confers a primed state against *Pst* DC3000 infection. (a) Relative expression of *PRT1* during a WT developmental time course. (b) Relative expression of *PR5* and *ADH1* in WT and mutants during a developmental time course. (c) Relative expression of pipecolic acid synthesis and EDS1/PAD4 signaling‐responsive transcripts in WT and mutant plants. (d) Quantification of pipecolic acid in untreated mature leaves. (e) *Pst* DC3000 growth 4 days after bacterial infiltration (10^6^ cfu/ml) in plants pre‐treated with MgCl_2_ (white bars) or *Pst* DC3000 *avrRpm1* (10^8^ cfu/ml) (black bars). Data represent means ± *SEM*. Statistical differences were analyzed by ANOVA followed by Tukey test (*p* < .05) or Student's *t* test *p < .05, **p < .01, ***p < .001

Our proteomic analysis revealed increased abundance of defense‐associated proteins in *prt1‐1* in the absence of an infection. Interestingly, age‐related differences were found in *PRT1* expression in WT plants, that reduced after the second week following germination (Figure 5a), that may suggest an increased stability of PRT1 substrates during late developmental stages. Recently, we showed an age‐related increase in the accumulation of defense genes in N‐degron pathway mutants *prt6‐1* and *ntaq1‐3* (Vicente et al., 2019). A comparable analysis revealed an earlier increase of the expression of *PR5* in *prt1‐1* compared with the WT (Figure 5b). In contrast, expression of hypoxia‐responsive genes such as *ADH1* is reduced with age in *prt6‐1* plants (Vicente et al., 2019). This was also observed in the *prt1 prt6* double mutant but not in *prt1‐1.*

The proteomic analysis in untreated *prt1‐1* plants revealed increased abundance of proteins involved in SAR (Figure 3b). Among those proteins differentially upregulated in *prt1‐1* was SARD4 (AT5G52810.1; ratio 2.46), an NAD(P)‐binding Rossmann‐fold superfamily protein, that has a fundamental role in SAR as part of the biosynthesis pathway of the systemic signal Pip and its hydroxylated form N‐hydroxy Pip (Ding et al., 2016; Hartmann et al., 2018). Pre‐treatment with Pip protects distal tissues against a subsequent *Pst* infection (Wang et al., 2018). Increased SARD4 protein levels in *prt1‐1* were not accompanied by changed *SARD4* transcript accumulation, nor by accumulation of *AGD2‐like defense response protein1* (*ALD1*), the other enzyme involved in Pip biosynthesis from L‐lysine (Hartmann et al., 2018) (Figure 5c), which may indicate that they are subject to post‐translational regulation. Recently, it was demonstrated that Pip biosynthesis is tightly regulated by EDS1/PAD4 signaling (Hartmann & Zeier, 2018; Hartmann et al., 2018). Increased abundance of EDS1 (and several EDS1‐responsive proteins) in untreated *prt1‐1* plants was accompanied by small changes in the accumulation of EDS1‐responsive transcripts, including *FLAVIN‐DEPENDENT MONOOXYGENASE 1* (*FMO1*), a key component of plant immunity (Bartsch et al., 2006; Koch et al., 2006; Mishina and Zeier, 2006) that catalyzes the hydroxylation of Pip, *CALMODULIN‐BINDING PROTEIN 60‐LIKEg* (*CBP60g*) and *ISOCHORISMATE SYNTHASE1* (*ICS1*) (Cui et al., 2017) (Figure 5c). Interestingly, untreated double‐mutant *prt1 prt6* leaves showed increased levels of Pip compared with the WT, while no differences were observed in the single mutants (Figure 5d), supporting the idea of a synergistic interaction between both PRT1 and PRT6 pathways. Reductases in addition to SARD4 are involved in the synthesis of Pip (Hartmann et al., 2018), which might dilute the effect of the elevated accumulation of SARD4 in *prt1‐1*. Together with the increased levels of this metabolite in *prt1‐1 prt6‐1,* this might indicate a post‐translational compensatory mechanism between PRT1 and PRT6. These data led us to analyze whether the *prt1‐1* phenotype resembles the primed state generated in WT plants once SAR is induced by the pathogen. First, we tested the impact of the SAR‐inducing avirulent strain *Pst* DC3000 *avrRpm1* in leaves of *prt1‐1*, *prt6‐1,* and *prt1 prt6*, and this analysis showed a slightly lower growth, but not statistically significantly different to the WT (Fig. S6). As expected, the growth of *Pst* DC3000 in distal tissues of WT plants locally pre‐treated with *Pst* DC3000 *avrRpm1* was reduced; however, the pre‐treated mutants *prt1‐1*, *prt6‐1,* and *prt1‐1 prt6‐1* showed a similar growth of the virulent strain in distal tissues than the WT, therefore, a smaller reduction in *Pst* DC3000 growth compared with non‐pre‐treated plants (Figure 5e). This, together with other data shown here demonstrating defense components constitutively activated in untreated *prt1‐1,* indicates that the protective effect exerted by the onset of SAR in WT plants is already present in this mutant. Furthermore, the fact that *prt1‐1*, *prt6‐1,* and *prt1‐1 prt6‐1* do not exhibit a further increased resistance after the pre‐treatment with *Pst* DC3000 *avrRpm1* might indicate that a mechanism operates to limit the onset of a SAR response once a certain threshold is reached, although further analyses should be carried out to verify this proposition.

## 4 DISCUSSION

Selective plant N‐degron pathways recognize proteins with specific Nt residues and target these for degradation (Gibbs, Bailey, Tedds, & Holdsworth, 2016; Gibbs, Bacardit, et al., 2014; Gibbs, Isa, et al., 2014; Varshavsky, 2019). Although N‐recognin activities for all Arg/N‐degron pathways in yeast and mammals are present in single proteins containing UBR domains, early during, or even before, the evolution of Archaeplastida, the ClpS domain was lost from plant orthologous sequences. Concomitantly, PRT1‐like proteins can be identified in genomes of green algae and land plants (Viridiplantae), but not in taxa outside these groups. This suggests that non‐Viridiplantae taxa represented within the Archaeplastida either do not contain recognition components for aromatic Nt residues, or that they contain novel activities unrelated to those so far identified. It is remarkable that PRT1 protein domains and organization bear no relationship to the type 2 binding site lost in PRT6‐like sequences in Archaeplastida. Presumably PRT1 evolved as a de novo solution to the proteasomal removal of substrates with Nt‐aromatic residues, indicating that this N‐degron pathway function is an essential component in plants, and that substrates with aromatic Nt residues are important for plant growth and development. It was recently shown using protoplasts that one substrate of PRT1 is the E3 ligase BIG BROTHER (BB), which is cleaved by the protease DA1 to reveal Tyr‐61 (Dong et al., 2017), although no phenotype of *prt1* related to the *bb* phenotype was shown, making the relationship between these two E3 ligases unclear. N‐recognin functions for other Nt‐destabilizing residues of the defined Arg/N‐recognin pathway (including L, K, H, and I) still remain to be discovered in plants, though it has already been shown that these residues are destabilizing in plants (Garzon et al., 2007; Graciet et al., 2010).

Here, we show, through molecular physiological approaches, that disruption of PRT1 activity alters the plant immune system, increasing understanding about the function of this elusive N‐recognin in plants. The *prt1‐1* mutation influences several aspects of the immune response related to SA biology, phytoalexins and the systemic response. It was shown in a previous report that *prt1‐1* was more susceptible to both *Pst* DC3000 and *Pst avrRpm1* (de Marchi et al., 2016), and it remains unclear why that phenotype was observed. Our results do not agree with those reported in that analysis, not only regarding the functions of *PRT1* but also *PRT6* and *NTAQ1*, as in a previous study we also observed increased resistance to *Pst* in *prt6* and *ntaq1* mutants. Furthermore, *prt6* displayed the same phenotype even when the analysis was performed under similar conditions for plant growth and infection used in de Marchi *et al.* (Vicente et al., 2019). Our analyses via independent approaches (including analyses of the *prt1* proteome and immune system‐related gene expression) clearly show constitutive upregulation of components of the immune response in untreated adult plants, which provides an explanation of the observed phenotype of enhanced tolerance to *Pst* DC3000. Importantly, these phenotypes were removed when PRT1 activity was restored to the *prt1‐1* mutant in transgenic plants containing tagged genomic *PRT1* sequence.

Interestingly, combination of *prt6 prt1* (thus removing N‐degron pathways for both type 1 and 2 Nt residues) led to enhanced accumulation of pathogenesis‐related proteins, metabolites and hormones in comparison with either single mutant. For example, it was recently reported that *prt6‐1* did not show increased levels of SA during infections (Vicente et al., 2019), whereas increased levels greater than those seen for *prt1‐1* alone were observed in uninfected *prt1‐1 prt6‐1.* In addition, accumulation of defense‐related protein PR2 was much greater in the double mutant than either single. Of particular significance, we observed increased SA and Pip levels in untreated *prt1‐1 prt6‐1* compared to WT. Consistent with this, we observed upregulation of SA‐responsive proteins and a key enzyme of Pip synthesis. Both have been shown to be important elements of local and systemic defense responses induced against *Pst* DC3000. These data clearly indicate a synergistic effect of both PRT1 and PRT6 activities in enhancing SA accumulation. A positive transcriptional feedback loop to reinforce the response during pathogenic infections cannot be ruled out.

Surprisingly, the tolerance of the double mutant to *Pst* was not statistically significantly different to that of single *prt6* or *prt1* mutants, which may happen if PRT1 and PRT6 control the stability of a common pool of substrates, by recognizing different types of N‐degrons in them. If that is so, those proteins still could be degraded by the other E3 ligase in the single mutants, and just removing both activities could result in a complete stabilization of those proteins. It can be also hypothesized that both activities affect the stability of proteins in different steps of common pathways. Furthermore, it cannot be ruled out that the lack of further pathogen growth restriction in the double mutant is due to the pleiotropic nature of these mutants in stabilizing all associated substrates (some of which might not be defense‐related proteins), the combination of which in the double mutant may interfere in the response to *Pst*. The importance of the N‐terminal regulation of the stability of defense proteins has also been illustrated recently in mammals. In this case, it was shown that N‐degron pathway‐regulated degradation of the N‐terminal of part of an NLR receptor that perceives pathogenic effectors, called NLRP1B, is actually the mechanism that triggers its activity (Chui et al., 2019; Sandstrom et al., 2019).

It has been demonstrated recently that the increased resistance to *Pst* displayed by the NTAQ1 branch of the PRT6 N‐degron pathway is camalexin‐dependent (Vicente et al., 2019). Here, we show that this phytoalexin pathway is also affected in *prt1‐1*, reinforcing the idea of a synergistic interaction between both branches. However, the decreased levels of JA/JA‐Ile, together with elevated SA/SAG, could lead to an increased susceptibility of these mutants to other pathogens, such as the necrotrophic bacterium *Pectobacterium atrosepticum*, the fungus *Alternaria brassicicola* or the biotrophic bacterium *Xanthomonas oryzae* (Zhang, Zhang, Melotto, Yao, & He, 2017). A modest increased accumulation of the protein SUPPRESSOR OF BIR1‐1 (SOBIR1; AT2G31880.1; ratio 1.61) in *prt1‐1* (Table S1) further reinforces the importance and complexity of the role of the PRT1 E3 ligase in plant defense. SOBIR1 is a receptor‐like kinase that works as a common adaptor for many receptor‐like proteins (RPLs), thus mediating RLP‐mediated resistance to a diverse range of pathogens (Takahashi, Murano, & Ishikawa, 2018; Zhang et al., 2014, 2013). One of these RPLs, RPL23 (AT2G32680.1; ratio 2.36), also showed increased accumulation in *prt1‐1*. Importantly, *prt1‐1* also showed increased accumulation of negative regulators of the defense response against *Pst*, such as NUDT7 (AT4G12720; ratio 2.29) and two 2‐oxoglutarate Fe(II)‐dependent oxygenases, DMR6 (AT5G24530; ratio 2.01) and DLO1 (AT4G10500; ratio 2.05), these last two with partially redundant activities. The increased amount of these proteins would be in line with their previously described function in lessening the induced defense response in order to avoid the self‐damage that a prolonged immune activation might cause in the plant (Ge et al., 2007; Zeilmaker et al., 2015). Therefore, this could be a part of a homeostatic feedback mechanism deployed in response to the pool of defense‐induced PRT1 substrate proteins over accumulated in *prt1‐1*. This, together with the fact that *prt1‐1* does not show the greatly increased levels of SA accumulation or SA‐dependent gene expression that autoimmune mutants (commonly dwarf) do, could explain why *prt1‐1* shows no growth arrest despite the enhanced activation of defense responses. Further analyses should be performed to verify other possible phenotypic changes associated with a constitutively activated defense such as microscopic cell death lesions. Nevertheless, results obtained in this work provide evidence for clear differences between roles of the two N‐recognin branches. Our observation that *ADH1* expression is not influenced by loss of PRT1 function argues against a possible connection between PRT1 and the regulation of plant oxygen sensing driven by PRT6 (Gibbs et al., 2011; Licausi et al., 2011). Furthermore, *PRT1* transcript levels decrease with age, which may indicate that PRT1 could be involved in a form of developmentally regulated defense called age‐related resistance (Carella, Wilson, & Cameron, 2015).

The PCO branch of the PRT6 N‐degron pathway has already been described as an active component of the plant defense mechanism. The stability of an artificial Nt‐Cys substrate was increased in leaves following inoculation of *Pst* or of the PAMP flg22, and known Nt‐Cys/PRT6 substrates ERFVIIs were shown to be involved in stomatal movement upon *Pst* perception, and in gall development during clubroot infection (Vicente et al., 2019, Gravot et al., 2016). In line with this, our data suggest that, together with PRT6, loss of PRT1 N‐degron pathway function induces components of the plant immune system, including the systemic response SAR. Great advances have been made in the last decades in the study of the generation, travel, and recodification in distal tissues of the systemic signal by characterizing several molecules responsible for this process (Lim et al., 2016; Wang et al., 2014; Yu et al., 2013). In parallel with the SA/MeSA signaling pathway, other systemic signal molecules including NO, ROS, AzA, and G3P work forming a linear pathway, and it has been proposed that Pip works upstream of this linear pathway at the primary site of infection. Therefore, increased levels of SA and Pip in *prt1‐1 prt6‐1* suggest that PRT1 and PRT6 influence both branches of the SAR.

Overall, these data suggest that PRT1, in addition to PRT6, could act to limit the onset of the plant defense response in the absence of a pathogen by constitutively degrading N‐degron substrates with type 2 Nt‐destabilizing residues responsible for local and systemic induction. Once a threat is perceived, reduced activity of these N‐recognins or cognate proteases (whose activity produces type 2 destabilizing residues as part of N‐degrons) may allow substrate or pre‐substrate accumulation and induction of the defense response. However, taking into consideration that the identity of almost all PRT1 substrates and related functions remain elusive at present, it is possible that the constitutive stabilization of PRT1 substrates not directly related with the plant immune system could perturb cellular homeostasis and influence the plant immune response indirectly. The identification of proteins whose stability is regulated by the PRT1 N‐degron pathway will provide the information needed to locate this mechanism within the plant immune system. Together with the already identified roles for PRT6, our findings indicate that protein substrates of these N‐degron pathways encode important functions related to the control of the immune response, with biotechnological potential.

## CONFLICT OF INTEREST

The authors declare no conflict of interest associated with the work described in this manuscript.

## AUTHOR CONTRIBUTIONS

J.V., H.Z., F.L.T., and M.J.H conceived the original research plans; J.V., H.Z., F.L.T., K.S.L., R.R., and M.J.H supervised the experiments; C.J.T., J.V., H.Z., M.O., V.P., M.J.D. performed experiments; J.V., H.Z., V.P., F.L.T., and M.J.H designed the experiments and analyzed the data; J.V., F.L.T., and M.J.H wrote the article with contributions of all the authors; M.J.H. supervised and completed the writing. H.Z. is responsible for distribution of plasmids and plant materials. J.V. agrees to serve as the author responsible for contact and ensures communication.

## ACCESSION NUMBERS

At3g24800 (PRT1); AT3G57260 (PR2); AT1G09080 (MED37B); AT2G43570 (CHI); AT1G75040 (PR5); AT5G10760 (AED1); AT5G59670; AT3G57240 (BG3); AT1G02920 (GST7/GSTF6); AT2G14610 (PR1); AT2G04450 (NUDT6); AT2G44290; AT5G20230 (BCB); AT2G18660 (PNP‐A); AT5G18470 (CAN2); AT4G23140 (CRK6); AT5G52810 (SARD4); AT2G30140 (UGT87A2); AT2G31970 (RAD50); AT5G12940; AT2G32680 (RLP23); AT4G30930 (RPL21M/NFD1); AT1G30900 (VSR6); AT1G21250 (WAK1); AT4G32610 (MED37D); AT5G02490 (HSP70‐2); AT4G12720 (NUDT7); AT3G60420; AT1G32940 (SBT3.5); AT3G52430 (PAD4); AT1G02930 (GST6); AT1G13470; AT2G05380 (GRP3S); AT5G02780 (GSTL1); AT4G25000 (AMY1); AT1G26380 (FOX1); AT1G33960 (AIG1); AT4G10500 (DLO1); AT2G47130 (SDR3); AT3G48090 (EDS1); AT5G03350 (LLP1; AED9); AT4G05020 (NDB2); AT5G24530 (DMR6); AT1G65790 (RK1); AT5G11920 (CWINV6); AT5G20250 (DIN10); AT1G22530 (PATL2); AT4G09160 (PATL5); AT5G05890 (UGT76C5); AT4G30270 (XTH24); AT3G25760 (AOC1); AT3G16420 (PBP1); AT2G23120; AT3G15950 (NAI2); AT5G61160 (AACT1); AT2G45180.

## LARGE DATASETS

Mass spectrometry proteomics data have been deposited to the ProteomeXchange Consortium via the PRIDE partner repository (Vizcaino et al., 2016) with the dataset identifier PXD014238.
