# Supplementary material for: The Arabidopsis thaliana N‐recognin E3 ligase PROTEOLYSIS1 influences the immune response
Source: Plant Direct. 2019 Dec 26;3(12):e00194. doi: 10.1002/pld3.194 (PMC6933115; doi:10.1002/pld3.194)
Supplement: Supplementary file 1 [file PLD3-3-e00194-s001.pdf]

Figure S1

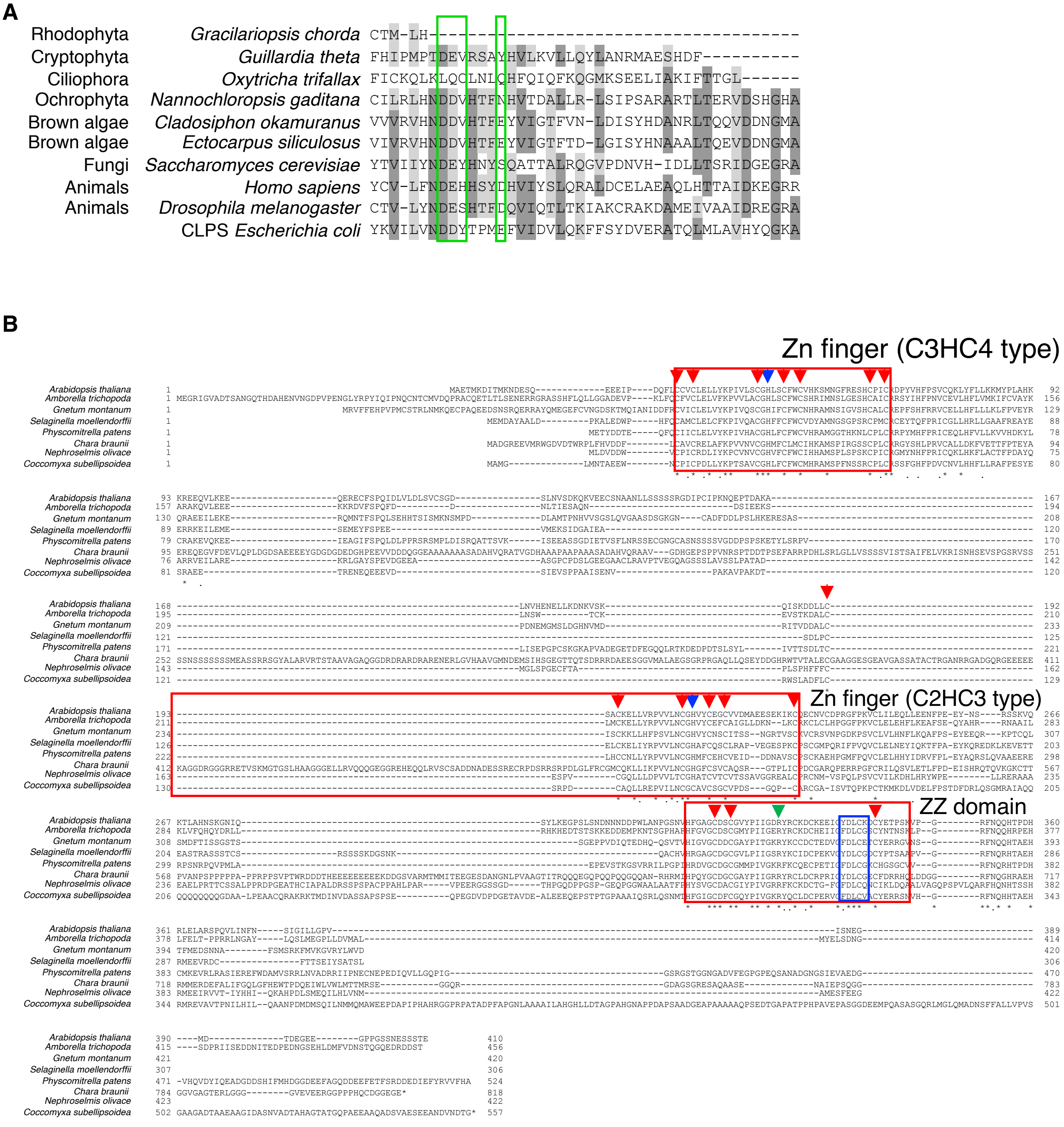

Figure S1 I Analysis of the evolutionary history of PRT6 and PRT1 (A) Proteins aligned via MUSCLE from selected species with the ClpS domain and three without from within Algae-like taxa, fungi, animals and the ClpS domain of *E. coli*. Shading: amino-acid similarity; dark shading is >50% identity; green boxes surface-exposed residues in ClpS; **(B)** MUSCLE alignment of PRT1 related sequences from plants. *= identical, .= similar residues; red, blue and green arrow heads: conserved Cys, His and Arg residues; red boxes: positions of Zn finger and ZZ domains; blue box: the core ZZ sequence.

Figure S2

A

MAETMKDITMKNDESQEEEIPDQFLCCVCLELLYKPIVLSCGHLSCFWCVHKSMNGFRESHCPICRDPYVHFP
SVCQKLYFLLKKMYPLAHKKREEQVLKEEQERECFSP**Q**IDLVLDLSVCSGDSLNVSDKQKVEECSNAANLLSS
SSSRGDIPCIPKNQEPTDAKALNVHENELLKDNKVSKQISKDDLLCSACKELLVRPVVLNCGHVYCEGCVVDM
AEESEKIKCQECNVCDPRGFPKVCLILEQLLEENFPEEYNSRSSKVQKTLAHNSKGNIQSYLKEGPSLSNDNNN
DDPWLANPGSNVHFGAGCDSCGVYPIIGDRYRCKDCKEEIGYDLCKDCYETPSKVPGRFNQQHTPDHRLELA
RSPQVLINFNSIGILLGPVISNEGMDTDEGEEGPPGSSNESSSTE
*Q111-STOP ***prt1-1***

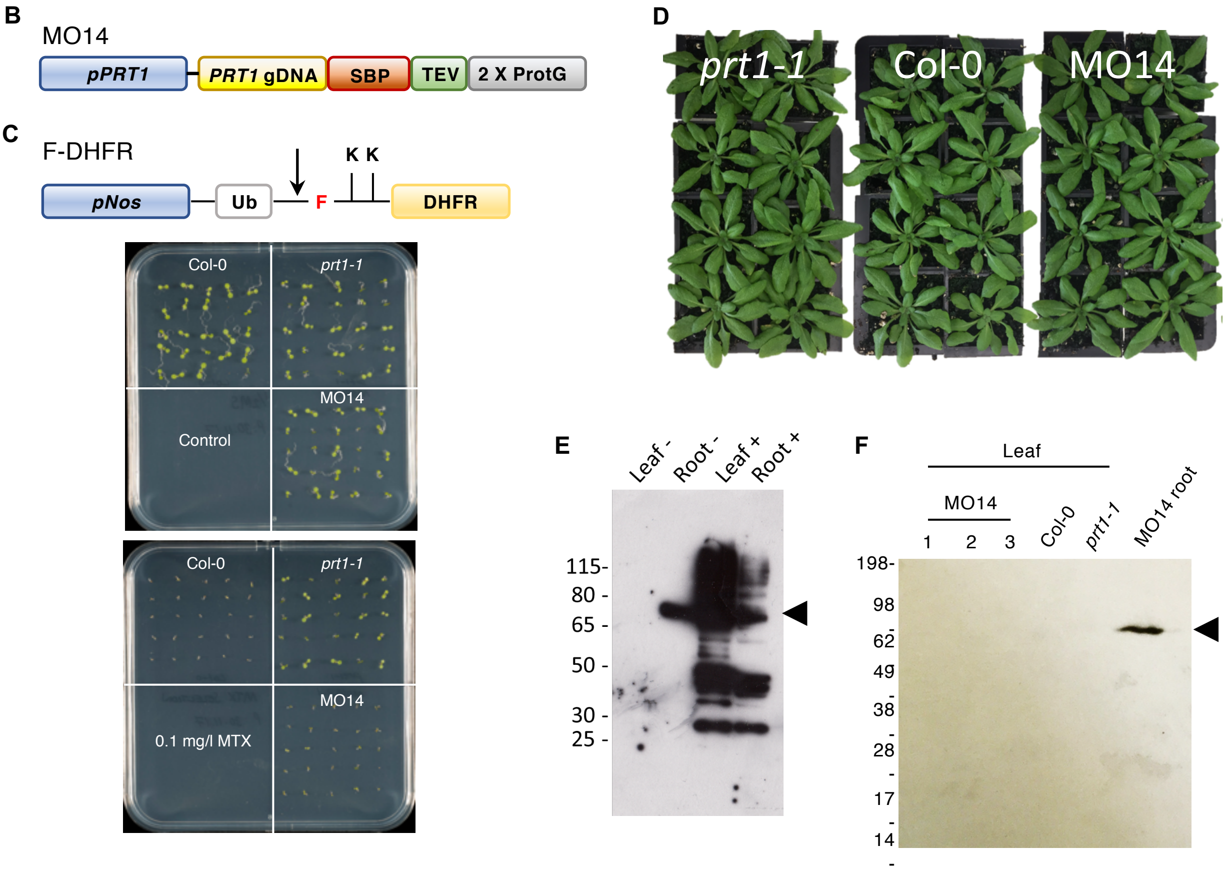

Figure S2 I Complementation of the *prt1-1* mutation. (A) PRT1 protein sequence indicating SNP of *prt1-1* allele. **(B)** Schematic of the PRT1 transgene (MO14) used for complementation of *prt1-1*. *pPRT1*, *PRT1* endogenous promoter; *PRT1 gDNA*, full length *PRT1* genomic DNA; SBP, streptavidin binding protein; TEV, tobacco etch virus cleavage site; ProtG, immunoglobulin G binding domain (IgG-BD) of Protein G. **(C)** Complementation of *prt1-1* mutation on methotrexate (MTX). Seeds of the indicated genotypes were plated on 1/2MS or 1/2MS containing 0.1mg/L methotrexate hydrate and incubated for 5 d in constant light following 2 d of stratification. Scale bar = 10 mm. *prt1-1* plants contain the Ub-F-DHFR transgene shown in the schematic (Bachmair *et al.*, 1993). *pNOS*, nopaline synthase promoter; Ub, ubiquitin, DHFR, mutant variant of mouse dihydrofolate reductase with reduced affinity to MTX, preceded by a short linker containing lysine residues (K) that permit ubiquitylation. Cleavage of this fusion protein by endogenous deubiquitylating enzymes (indicated by the arrow), releases DHFR with an N-terminal phenylalanine residue (F). This F-DHFR protein is stabilized in *prt1-1* and confers resistance to MTX. Restoration of PRT1 function in the complementing line results in wild-type sensitivity of plants to MTX, due to degradation of F-DHFR. **(D)** Phenotypes of 4-week-old WT, *prt1-1* and MO14 plants grown in soil. **(E)** Immunoblot of crude proteins extracted from 7 day old seedlings expressing *pPRT1::gPRT1-GSTag* (MO14), treated with 50 μM Bortezomib (+) or vehicle (-). The blot was probed with α-SBP Tag antiserum. **(F)** Immunoblot of crude proteins from 28 d old leaves of different genotypes. Proteins from 5 d old roots are included as a positive control. The blot was probed with α-SBP Tag antiserum. For **(E)** and **(F)**, blots are representative of two independent experiments and positions of Mr markers (kDa) are indicated to the left of the blot. Expected size of PRT1-Tag (indicated by arrowhead) is ~71 kDa.

Figure S3

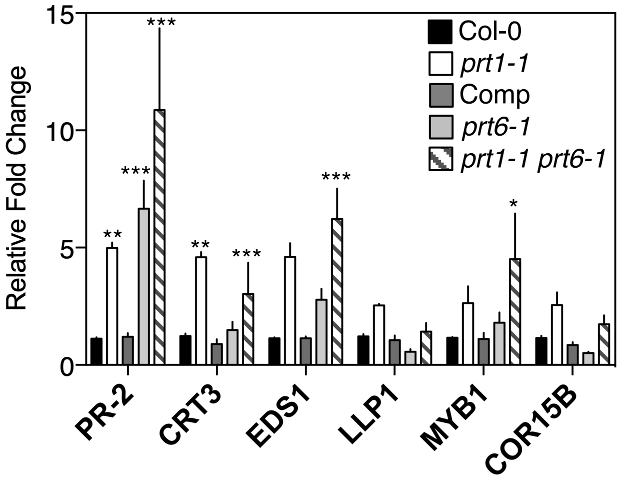

Figure S3 | Analysis of RNA expression for proteins identified as up-regulated in *prt1-1* compared to WT proteomics. Data represent means ± SEM. Statistical differences were analyzed by Student's t-test *p <0.05, **p < 0.01, ***p < 0.001.

Figure S4

Figure S4 I Comparison of proteins differentially regulated in *prt1-1* to public transcriptome data. The images were created using the Signature function of Genevestigator (Hruz et al., 2008) and shows transcriptome patterns (A) most similar and (B) most different to that of the *prt1-1* upregulated proteome. The proteome signature comprises proteins with ≥ 2-fold change in abundance at p ≤ 0.05, which are represented by at least two unique peptides.

A

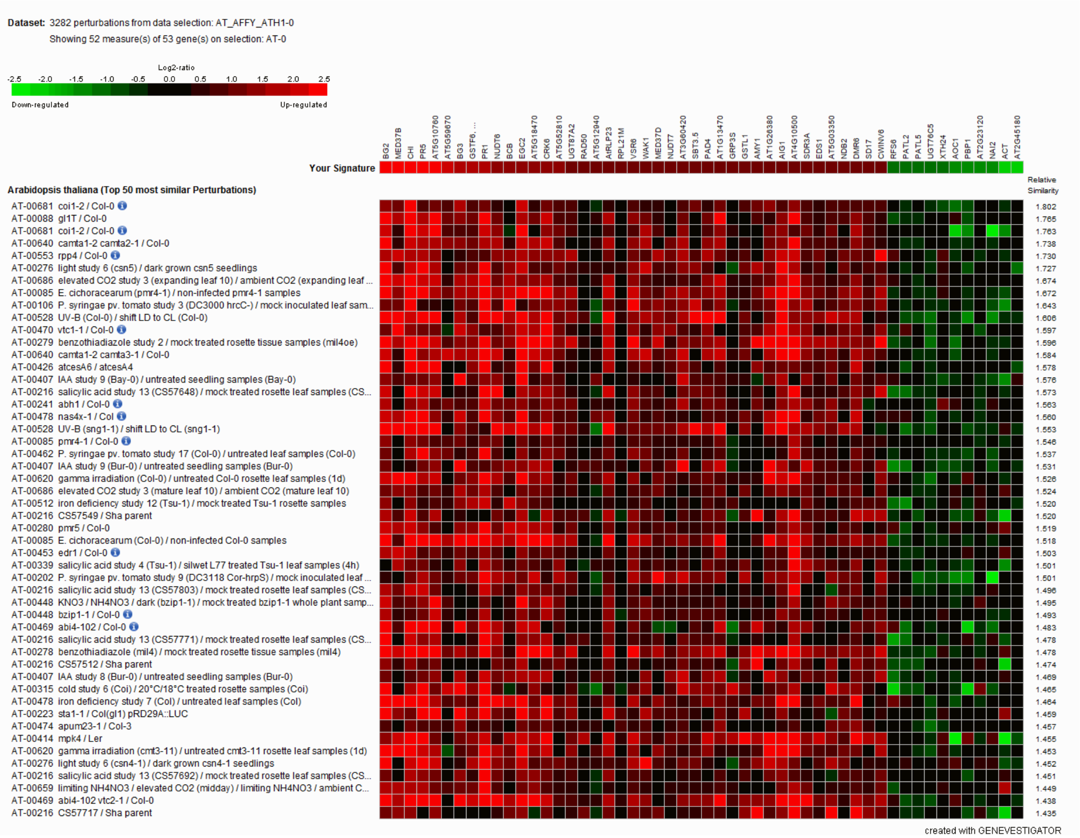

B

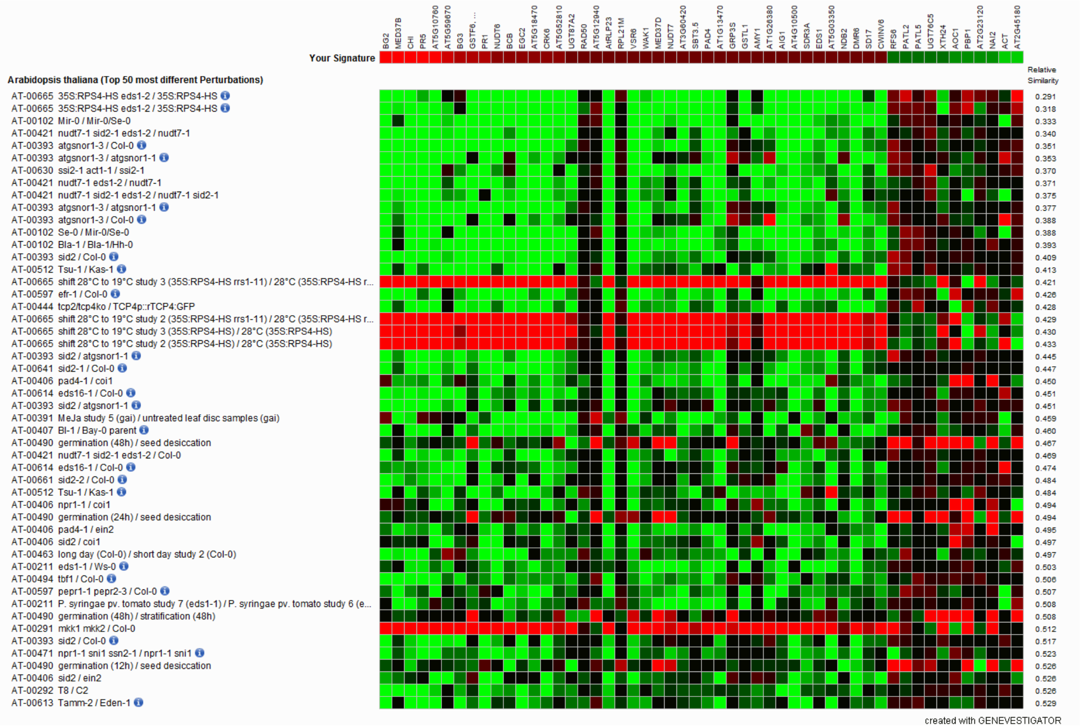

Figure S5

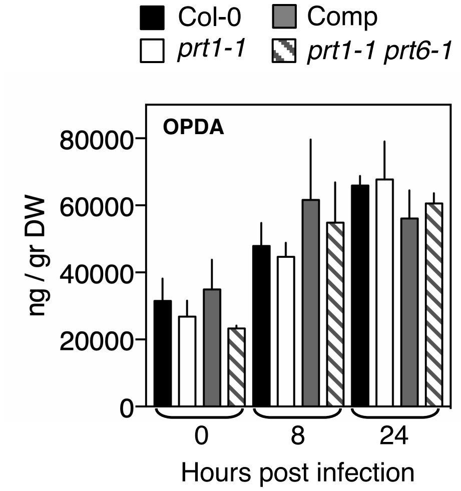

Figure S5 l Time course quantification of OPDA (12-oxo-phytodienoic acid) levels in response to *Pst* DC3000 infiltration of mature leaves in *prt1-1,* complemented *prt1-1, prt1-1 prt6-1* and WT. Data represent means ± SEM. Statistical differences were analysed by Student's t-test.

Figure S6

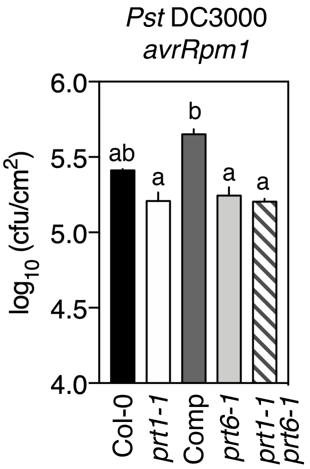

Figure S6 | Quantification of *Pst* DC3000 *avrRpm1* (10^6 cfu ml^{-1}) growth 4 days after injection. Data represent means ± SEM. Statistical differences were analyzed by ANOVA followed by Tukey test ($P < 0.05$).
